# Microprocessor–based monochromator controller

**DOI:** 10.1155/S1463924680000272

**Published:** 1980

**Authors:** Richard Dalle-Molle, James D. Defreese

**Affiliations:** University of Kansas Department of Chemistry Lawrence Kansas 66045 USA


### Microprocessor-based monochromator controller

**Richard Dalle-Molle and James D. Defreese***
*University of Kansas, Department of Chemistry, Lawrence, Kansas 66045, USA.*

### Introduction

The modular nature of the EU-700 series spectrometers (GCA/McPherson Instrument, Acton, Mass.) makes them an ideal choice for use as building blocks in the research laboratory. The fact that the instrument can be read and controlled in the digital domain is another advantage for those desiring laboratory-built spectrometric apparatus.

The EU-700 monochromator is normally coupled with the EU-700-32 Controller [1]. The monochromator performs its various functions by receiving digital control signals from this controller. An operator may slew or step the monochromator in the direction of either increasing or decreasing wavelength. Linear-in-wavelength scans may be carried out in either direction at six predetermined scan rates. The controller does not compensate for the wavelength errors caused by lag in the mechanical components of the device when changing scan direction. The wavelength scanning is controlled in an open loop manner, i.e., the controller sends a predetermined number of digital pulses to the monochromator's stepper motor but, however, it does not check that the monochromator has actually moved the correct number of wavelength increments.

Features of the present system include closed loop control of wavelength setting for improved accuracy and precision, a bidirectional scan capability, and a wavelength programming capability (e.g., scans linear in energy or wavelength, stepping between discreet wavelengths, etc.). This controller can function in a stand-alone mode in which interaction with the operator is through a keyboard, or as an "intelligent" monochromator when put under the control of another computer.

The microprocessor was chosen in preference to other types of "hardware" controllers for a number of reasons; it is easier to implement, easier to modify the design and it is more adaptable to changing requirements in monochromator control. An interrupt-based microprocessor controller for GCA/McPherson monochromators has been described by Lovse [2].

The system developed by the authors operates generally in the following manner. The controller accepts two commands, indicated by the ASCII characters "C" (Calibrate) and "M" (Move). Following the "C" command, the controller accepts a 5-digit decimal ASCII number, which is interpreted by the controller as the present wavelength setting of the monochromator. Once the controller has this number, it is "calibrated". The user may now enter the "M" command followed by a 5-digit decimal ASCII number. This number is interpreted as the wavelength to which the monochromator must "move". The present and desired wavelength are compared, and the magnitude and direction of the difference between them are calculated and stored. If the move indicated is greater than 10 nm (1000–0.01 nm steps) the slew motor is engaged and the monochromator moves in the appropriate direction. TTL level pulses from the monochromator's optical encoder (at 0.01 nm intervals) are counted by the controller, and when the monochromator has moved to within 200 steps of the desired wavelength, the slew motors are disengaged and a stepper motor is engaged. Pulses continue to be counted until the desired wavelength is reached. The stepper motor is disengaged, but the controller continues to monitor the optical encoder for extra pulses for a period of about 400 msec. At the end of this time, the number of pulses are checked once more, and if the monochromator is at the desired wavelength, this wavelength is given the status of the "present" wavelength. Control is returned either to the user if under keyboard control or to another software routine if the controller is under the management of another computer. The "calibrate" command is required only in the initial set up procedure; thereafter, the "move" command is used since the monochromator remains calibrated at the end of each move.

*Address correspondence to this author

### Instrumentation

Figure 1 is a complete circuit diagram for the microcomputer portion of the controller, the heart of which is an Intel (Santa Clara, CA) 8085 microprocessor [3]. Communication between the monochromator and the controller is handled through the parallel I/O ports of an Intel 8155 RAM–I/O–Timer chip [3]. The 8155 chip also contains sufficient read/write memory to store the variables associated with monochromator control and a programmable timer which is used to generate the necessary waveforms to control the monochromator's stepper motor. Communication between the controller and keyboard or another computer is handled via the SID (serial in data) and SOD (serial out data) pins of the 8085. The control program is stored on a 2708 IK-byte programmable read-only memory (PROM).

The 8212 8-bit latch is used to make the 2708 PROM compatible with the multiplexed address/data bus ($AD_0$ – $AD_7$) of the 8085 [4]. When $\overline{CS}$ of the 2708 is low, the chip is selected. The 10 bits of address necessary to select a given memory location in the 2708 are supplied by lines $AD_0$–$AD_7$ and $A_8$–$A_9$ from the 8085 chip. During the memory read cycle the function of $AD_0$–$AD_7$ changes such that $AD_0$–$AD_7$ now becomes the 8085's data bus. Address latch enable (ALE) is used to latch the address from the 8085 just prior to the change in function of $AD_0$ –$AD_7$. The address presented to $A_0$–$A_9$ of the 2708 thus remains valid while outputs $O_0$–$O_7$ present their information to the (now) data bus of the 8085. Similar circuitry is incorporated into the 8155 and it is not necessary to latch the address information externally.

Figure 2 shows the interface circuitry required to link the microcomputer to the monochromator. The signals which engage the monochromator slew motor are output from parallel I/O port B ($PB_x$) of the 8155. A logic 1 at $PB_0$ causes the slew motor to be engaged and to move the monochromator from low wavelength to high wavelength. A logic 1 at $PB_1$ causes a similar action, the monochromator slewing from high wavelength to low wavelength. If a logic 1 occurs simultaneously at $PB_0$ and $PB_1$, no action occurs.

The 4-phase waveforms required to move the stepper motor are generated by the timer output of the 8155 and a 74LS193 up/down counter. The direction of stepper motor movement is controlled by the output bit designated $PB_2$.

This bit controls the flow of the clock signal from the timer to either the count-up or count-down input of the up/down counter. This in turn causes the 4-phase signals to be generated for the stepper motor movement in the correct direction, either increasing or decreasing wavelength.

The 8155 timer is used as a programmable down counter which gives an output pulse each time the counter reaches zero. Therefore by varying the count loaded into the counter register, it is possible to vary the rate at which the stepper motor moves. Signals from the upper and lower wavelength limit switches of the monochromator are used to disengage the slew or stepper motor in the event that one of the limits is reached.

At each 0.01 nm step of the monochromator, the optical encoder within the device puts out a short TTL level pulse. Separate pulses are generated for upward and downward movements of the monochromator. Although the movement of the slew or stepper motors is only in one direction at a given time, bounce in the mechanisms causes occasional movements opposite to the general direction of the move. It is therefore necessary to sense the direction of the movement indicated by each encoder pulse. Up and down pulses are therefore brought into the controller separately. The up and down pulses are brought into $PA_0$ and $PA_1$ of port A on the 8155. These lines are also routed to a 74121 monostable chip. Any pulse which occurs generates an output pulse from the monostable which goes to $PC_2$ of port C. This bit is software programmed as a strobe for the input to port A. Each pulse from the optical encoder generates a strobe pulse which latches the optical encoder information into port A. Bit $PC_1$ acts as a port A buffer full flag. The controller reads port C until $PC_1$ indicates that the port A buffer is full. When this occurs port A is read. After port A has been read it is automatically reset by the 8155.

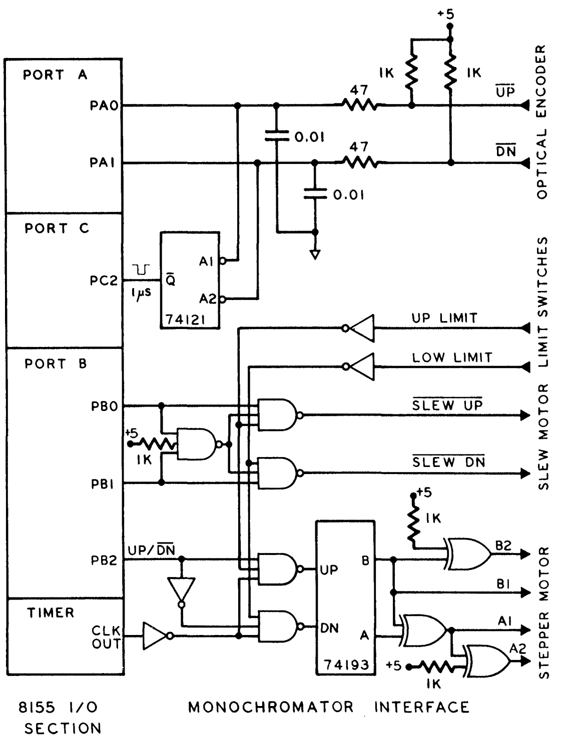

*Figure 2. Interface between microcomputer and monochromator.*

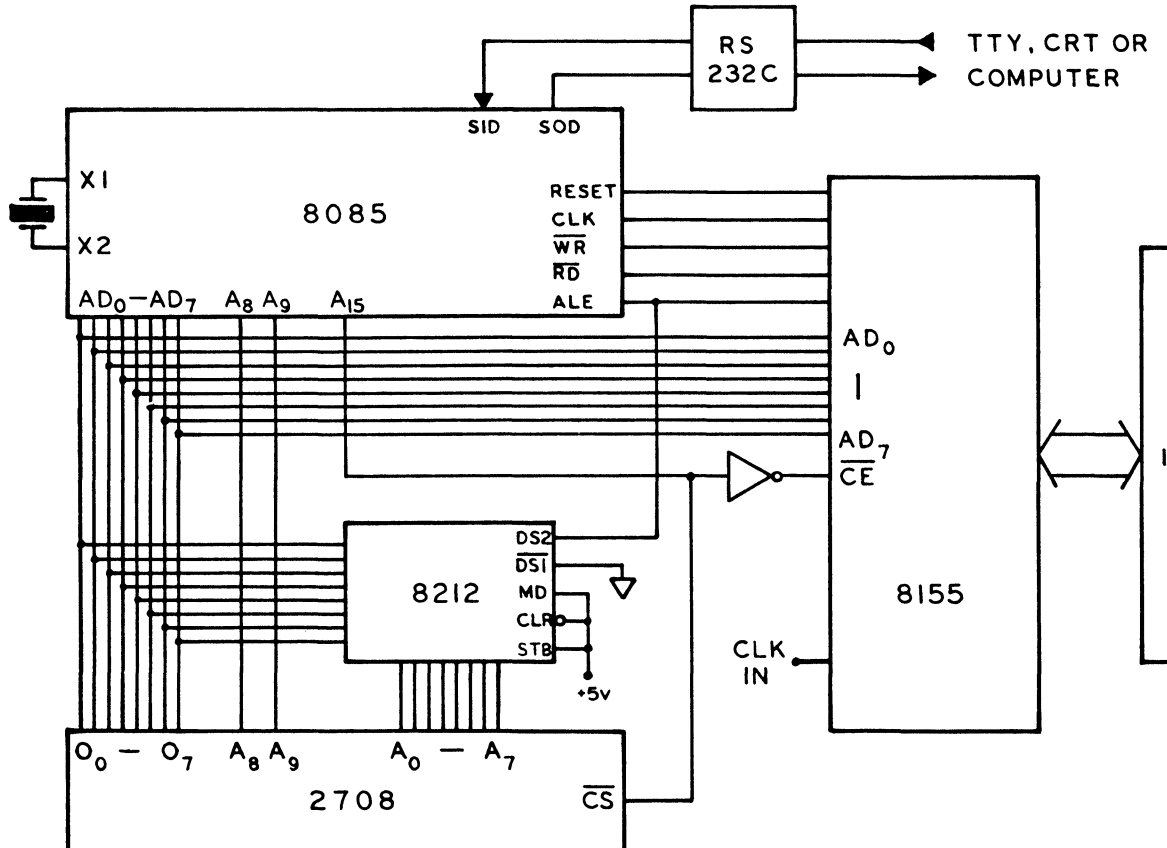

*Figure 1. Circuit diagram for microcomputer section of monochromator controller.*

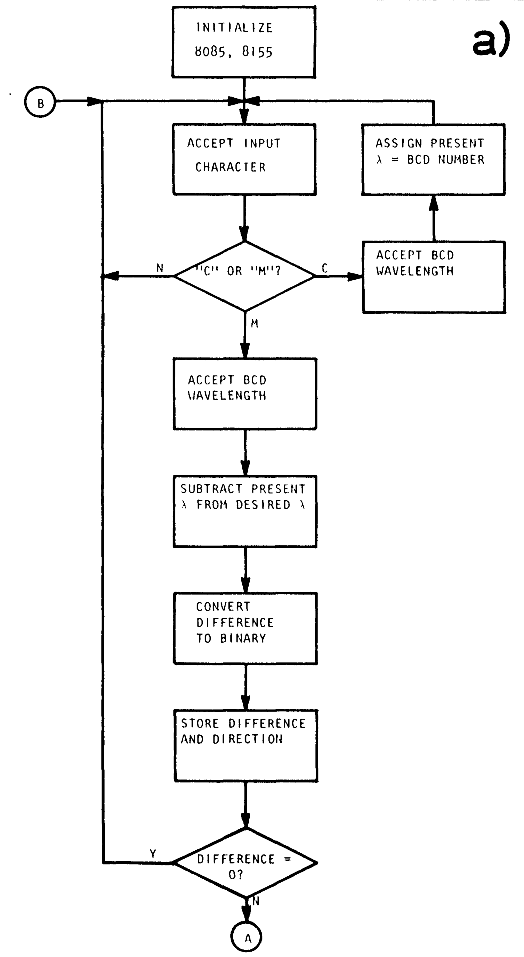

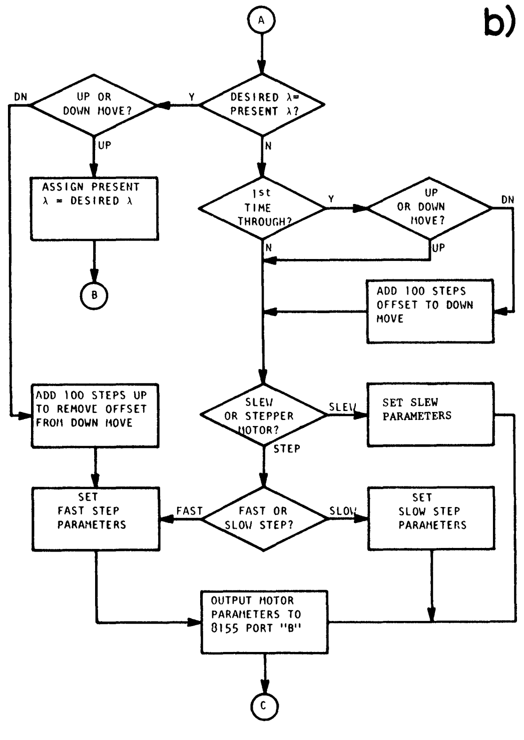

The contents of port A indicate whether an up movement or down movement has occurred, and this information is used to increment or decrement a counter in software. After each encoder pulse, the counter is checked against the desired number of counts for the move in progress. This information is then used to decide whether the movement should continue as it is, or whether it is time, for example, to change from the slew motor to the stepper motor. When the difference between the desired number of steps and the number in the software counter is zero, all motors are disengaged. Since the inertia of the motors may carry them a step or two past the desired setting, the controller continues to monitor the optical encoder for a period of ∿ 400 msec after the motors have been disengaged. If necessary the controller may re-engage the motors.

### Software

The software for the controller is written in Intel 8080 assembly language, which is compatible with the 8085 microprocessor with the exception of the "SIM" and "RIM" commands which were handled in EQU statements. The program is 928 bytes in length and is stored in a 2708 1K EPROM. The EPROM was programmed in the laboratory on a Cromemco (Mountain View, CA, USA) "Bytesaver" board. The 8155 contains 256 bytes of read/write memory. Thirty bytes of this memory are used for storage of flags and intermediate results and the rest is available to provide a processor stack memory.

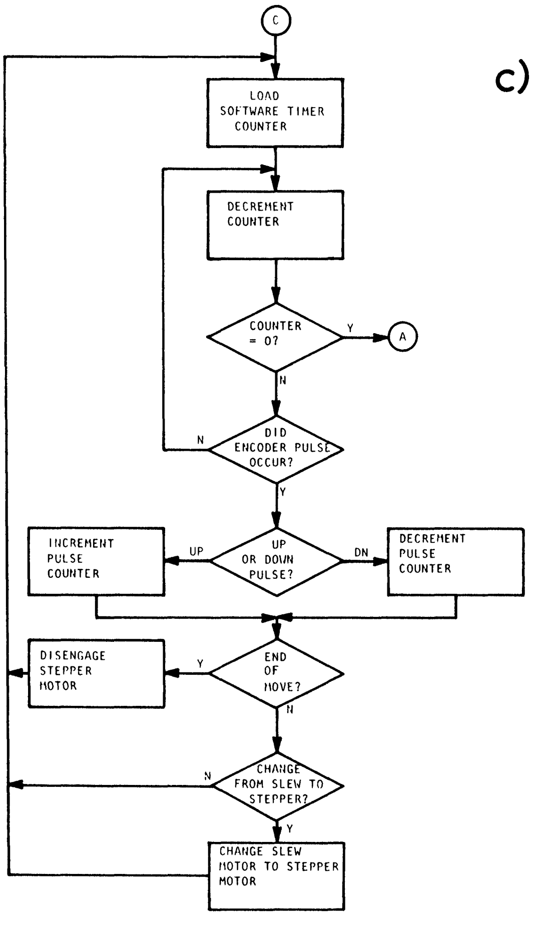

*Figure 3. Flowcharts for the controller software; a) initialization and user I/O routine, b) slew or stepper motor selection, and c) optical encoder pulse counting routine.*

The controller software is divided basically into 3 sections. These are depicted schematically in the flowcharts of Figures 3a–c.

The first section of the software (Figure 3a) contains all the initialization and user I/O routines. At system turn-on, the processor stack is set, the 8155 I/O port functions are programmed and the I/O lines of the 8085 are enabled. The controller then calls a baud rate identification routine [5,6]. The user (or managing computer) outputs a "space" character (20H) and the serial bit pattern is used by the controller to determine the baud rate of the terminal with which it is communicating. The SID and SOD pins of the 8085 are used to form serial communication links between the controller and other devices. It is possible in this fashion to communicate at baud rates from below 110 baud to greater than 9600 baud. Having identified the baud rate, the controller responds with a prompt character, a ">" is used.

The controller now proceeds to a keyboard reader routine which accepts input commands. The "C" and "M" commands are accepted, followed by the 5-digit wavelength as explained earlier. The "RUBOUT" command is also supported and allows the user to correct mistakes entered at the keyboard. If the input is specified as calibration, the input information is stored as the present wavelength in packed BCD representation. The controller then outputs a prompt character and awaits the next command.

When a move is specified, the input information is stored as the desired wavelength. The controller software subtracts the present wavelength from the desired wavelength and stores the result. This number, equal to the number of steps necessary for the move, is converted to binary and is also stored. The direction of the move (increasing or decreasing wavelength) is determined in the subtraction routine and is stored as a single bit flag.

The second section of the program (Figure 3b) selects whether the slew or stepper motor is to be used for the move. If the move is more than 20 steps but less than 1000, the stepper motor will be engaged and the 8155 timer section will be programmed to produce a clock rate which generates a step speed of 200 steps/second. This is the maximum recommended speed for the monochromator's stepper motor. A slower step speed is used for moves of less than 20 steps.

Mechanical lag in the leadscrew and sine-bar assembly cause sufficient inaccuracy in the decreasing-wavelength scans to make this type of scan inadvisable. Therefore the EU-700 is normally scanned in the direction of increasing wavelength. The controller has been programmed to always approach the final wavelength in the direction of increasing wavelength. Moves to lower wavelength are augmented in software with 100 additional steps. When this move is completed, the controller automatically sets the parameters for a move of 100 steps to higher wavelength. Thus a move from 500.00 nm to 450.00 nm is accomplished by first moving to 449.00 nm and then moving from 449.00 nm to 450.00. This approach ensures accuracy and precision independent of the direction of the move.

Once the parameters for motor speed and direction have been output, the program jumps to the optical encoder pulse counting routine defined in the third software section (Figure 3c). When a pulse has been latched at port A, it is

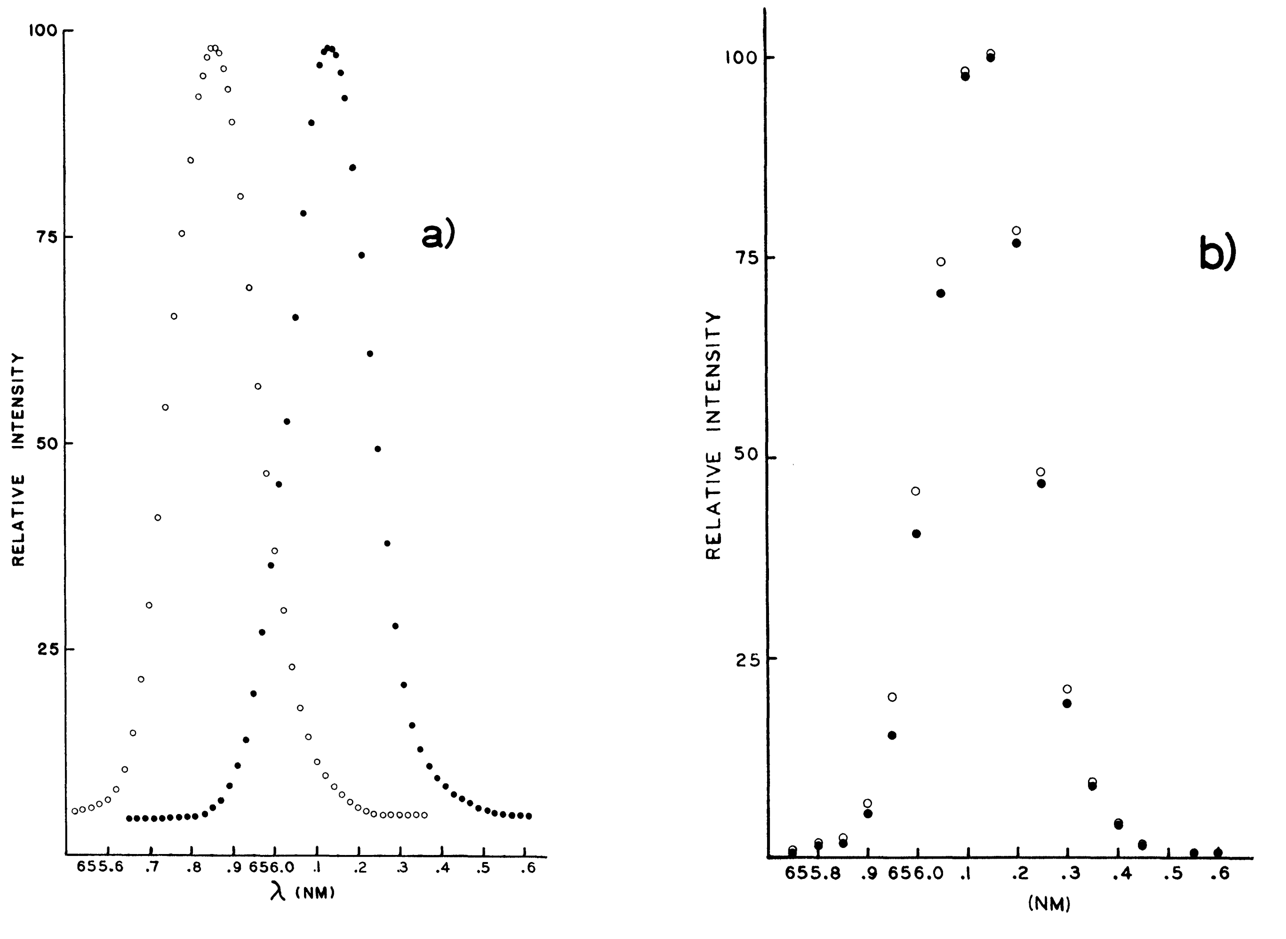

*Figure 4. Bidirectional scans of deuterium lamp emission line at 656.1 nm – (●) increasing wavelength direction, (o) decreasing wavelength direction; a) commercial controller, b) microprocessor-based controller.*

checked to see if it is an increasing or decreasing wavelength move. A software counter is incremented or decremented, depending upon the direction, and the counter is then compared to the desired number of steps for the move in progress. The remaining number of steps is also checked to see if a change from slew motor to stepper motor is required. This ensures that the new wavelength is approached smoothly.

The pulse counting routine contains a simple software timer which is implemented simply by loading a number into one of the CPU registers and decrementing it. Each time the register is decremented, the software checks $PC_2$ to see if a pulse has been latched. If no pulse has occurred, the register is further decremented and $PC_2$ is checked again. If a pulse has occurred, the above-mentioned pulse book-keeping is performed. The software timer register is then reloaded and the process begins again. When the desired and actual pulse counts are equal, the stepper motor is disengaged, but the pulse count routine reloads the software timer register and continues checking for pulses which might occur just after the motor is disengaged. This timer is set to count down in $\simeq$ 400 ms. If a pulse occurs during this time, it is handled in normal fashion and the counter is reset. Any 400 msec time frame in which no pulse occurs is considered as the end of the move. The pulse count routine makes a final tally of the actual and desired number of steps for that move, and passes this information back to the motor selection routine. If any additional steps are necessary, the motor routine may re-engage the correct motor and the pulse count routine is re-entered. If the actual and desired wavelength are equal, the desired wavelength is given the status of the present wavelength and the program returns to its monitor routine to await further commands.

### Results and discussion

Figure 4 shows the controller's ability to perform bidirectional scans while maintaining wavelength accuracy. The $D_2$ emission line at 656.1 nm was scanned in both increasing and decreasing wavelength directions with both the EU-700-32 controller and the microprocessor controller. Figure 4a shows the two scans with the EU-700-32 controller. The increasing wavelength scan is accurate, but because of the lag in the wavelength mechanism of the monochromator the decreasing wavelength scan is shifted by almost 0.3 nm. Although this difference will not be significant for most band spectra, it is quite disturbing when working with line spectra at bandpasses less than 1 nm. Figure 4b shows the same line scanned in the same manner using the present controller. The wavelength shift in this case cannot be distinguished. Although the exact agreement in peak wavelength in Figure 4b is somewhat fortuitous, in no case has it been found that the bidirectional scans to differ by more than 0.03 nm.

While the controller may be used by an operator at a keyboard, its real utility is more evident when it is placed under the management of another computer. Since the managing computer can be programmed to direct the controller to go to any wavelength in any order, it is possible to execute "wavelength programs". One may cause the monochromator to scan in linear energy units, for example, by calculating the wavelength equivalent for a given energy increment and outputting this value as a move to the controller. Scans carried out with the aid of the controller may be made at any speed (consistent with the hardware limitations), incorporating the necessary delays to allow signal averaging, changing over from one solution to another, etc.

The monochromator controller has proven to be reliable and accurate in laboratory use. Hardware design and troubleshooting were very straightforward. The major investment in time was in the development of software, a situation which will be encountered more often as microprocessors are increasingly applied in chemical instrumentation. A copy of the controller software is available on request.

The incorporation of inexpensive, dedicated microprocessor devices in chemical instrumentation is part of the general trend toward more highly automated chemical analysis. The relatively small price for hardware makes devices like the monochromator controller feasible. This clearly points towards the future when an instrument will have microprocessor control of each functional subunit. In the present case, the microprocessor controller has provided greatly increased flexibility in the use of any spectrometer which incorporates this particular controlled monochromator.

ACKNOWLEDGMENT

This research was supported by University of Kansas General Research Allocations #3095-XO-0038 and #3140-X038.

REFERENCES

[1] GCA Corporation, "700 Systems Instruction Manual – Model EU-700-32 Controller," Acton, Massachusetts, USA, 1975.
[2] Lovse, D., Ph.D. Thesis, University of Illinois, Urbana, Illinois, USA, 1977.
[3] Intel Corporation "MCS-85 USER'S Manual," Santa Clara, California, USA, 1978.
[4] Larsen D.G., Rony, P.R., Titus, J.A., and Titus, C.A., *American Laboratory*, 1978, **10** (9), 88.
[5] Ref. 3, p. A1 - 32.
[6] Ref. 3, p. A1 - 49.

